# Mapping the Spatial Sensitivity of Aquitard Hydraulic Parameters on Pumping Test Drawdowns

**DOI:** 10.1111/gwat.70014

**Published:** 2025-08-25

**Authors:** Martijn D. van Leer, Willem J. Zaadnoordijk, Alraune Zech, Jasper Griffioen, Marc F. P. Bierkens

**Affiliations:** ^1^ Department of Physical Geography Utrecht University Princetonlaan 8a 3584 CB Utrecht The Netherlands; ^2^ Geological Survey of the Netherlands TNO P.O. Box 80015 Utrecht 3508 TA The Netherlands; ^3^ Water Resources Section, Faculty of Civil Engineering and Geosciences Delft University of Technology Delft The Netherlands; ^4^ Department of Earth Sciences Utrecht University Princetonlaan 8a 3584CB Utrecht The Netherlands; ^5^ Copernicus Institute of Sustainable Development Utrecht University Princetonlaan 8a 3584 CB Utrecht The Netherlands; ^6^ Unit Subsurface and Groundwater Systems Deltares Daltonlaan 600 3584 BK Utrecht The Netherlands

## Abstract

This study investigates the spatial and temporal sensitivity of aquitard hydraulic conductivity and specific storage on drawdowns in pumping tests. The objective is to understand which area of the aquitard is represented by drawdowns in different observation wells. A three‐layered MODFLOW 6 model was used to simulate pumping tests on a circular Voronoi grid for three transmissivity scenarios and both confined and semiconfined top boundary conditions. A local sensitivity analysis was performed using PEST++ to determine how perturbations in hydraulic conductivity and specific storage of the aquitard affect head changes at observation wells in the pumped and overlying aquifer. Results indicate that for observation wells in the pumped aquifer, sensitivity forms an elliptical shape that is symmetrical around the observation well and the pumping well for all scenarios. The sensitivity map for the observation well in the overlying aquifer depends on the transmissivity ratio between both aquifers. It favors the area surrounding the pumping well if the transmissivity of the pumped aquifer is lower than that of the overlying aquifer. Conversely, with higher transmissivity in the pumped aquifer, sensitivity primarily lies around the observation well. Sensitivity patterns evolve over time, expanding the area of influence and shifting the sensitivity toward the observation well for a semiconfined top boundary. These findings are relevant for understanding the information regarding aquitard heterogeneity that is present in pumping test drawdowns and optimizing pumping test design.

## Introduction

Pumping tests are commonly used to determine hydraulic parameters of aquifers and aquitards. Typically, the parameters are identified using analytical solutions from drawdown measurements. The simplest solutions that consider aquitards only use observation wells in the pumped aquifer and include a hydraulic conductivity and specific storage value for confining layers (Hantush and Jacob 1955). However, these solutions account for leakage of the aquifer through both the overlying and underlying aquitards, resulting in a single value for both vertical flow directions, which leaves ambiguous results regarding the aquitards.

To achieve unambiguous interpretation of the aquitard hydraulic parameters, observation wells in surrounding aquifers must also be considered (Neuman and Witherspoon 1969, 1972; Hemker and Maas 1987). This requires pumping tests to be sufficiently long to let the drawdown propagate through the aquitard. Additionally, observation wells have to be installed in the overlying and/or underlying aquifer. This makes a pumping test more costly and requires more complex solutions for interpretation. Partly due to these challenges, aquitards are often neglected in favor of aquifers in pumping test design and interpretation (Fogg and Zhang 2016).

Analytical solutions for pumping test interpretation typically assume homogeneous hydrogeological units. However, this is not the case in reality. Multiple studies have investigated pumping tests assuming heterogeneity (Leven and Dietrich 2006; Zech et al. 2015; Demir et al. 2017; Müller et al. 2022; Manewell et al. 2023). These studies have focused on the heterogeneity of the pumped aquifers, without considering aquitards. The heterogeneity of aquitards during pumping tests has been considered from a leaky aquifer perspective with wells only in the pumped aquifer (Copty et al. 2008) and in an applied case through model calibration (van Leer et al. 2023). Little is known about the information to be gained from pumping tests regarding effective aquitard parameters. Furthermore, it remains unclear which area of the aquitard influences the effective aquitard parameters obtained and how they relate to the aquitard heterogeneity.

This study is the first to identify the spatial sensitivity of aquitard hydraulic conductivity and specific storage for pumping test drawdowns in the pumped aquifer and the aquifer above. This information is not only valuable to assess the heterogeneity of the aquitard, but is particularly relevant to optimize pumping test design for aquitard characterization. The latter is key information for solute transport studies and the parameterization of regional groundwater models.

## Methods

### Conceptual Model

**Figure 1:**
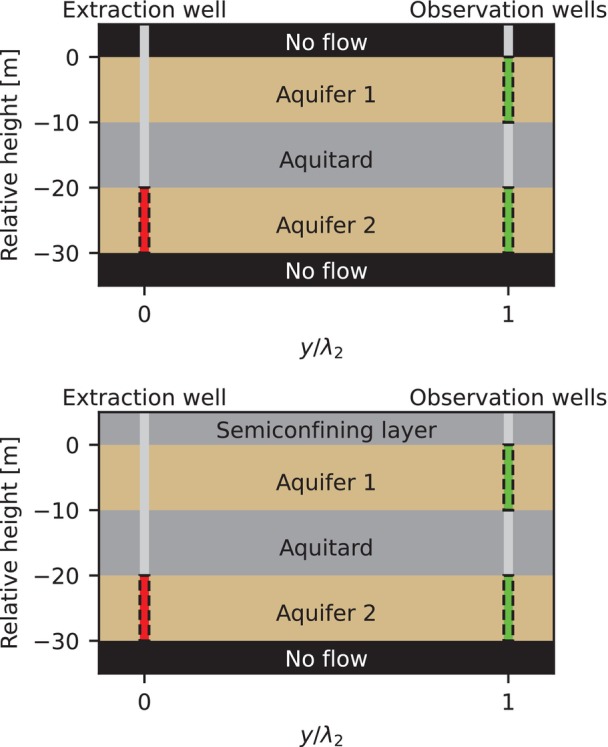
Conceptual representation of the three‐layer model used in the pumping test with (top) confined upper boundary and (bottom) semiconfined upper boundary layer.

We performed a sensitivity analysis using a numerical model that simulates a pumping test in a three‐layer setting. Figure 1 shows the conceptual model, which consists of two aquifers separated by an aquitard. All layers are of equal thickness D (L). The horizontal hydraulic conductivity in the aquifers is referred to as *K*_1_ (upper aquifer 1) and *K*_2_ (lower aquifer 2) (L/T). Vertical hydraulic conductivity in the aquifers is the same as the horizontal hydraulic conductivity. The aquitard is characterized by the hydraulic resistance c.

Two wells are placed: one pumping well which is pumping from the second aquifer and an observation well which has observation screens in both aquifers.

We test two situations for the upper boundary of the system: (1) a confined top representing a system not impacted by other layers and (2) a semiconfined top which represents settings with surface water or leaky aquitards.

### Dimensionless Variables

To make our findings generalizable and scalable, we use dimensionless variables. Neuman and Witherspoon (1969) described flow through an aquitard by five parameters: leakage factors $\lambda_{1}$ and $\lambda_{2}$ for both aquifers, leakage parameters $\beta_{1}$ and $\beta_{2}$ for both aquifers, and dimensionless time $t_{D}$. In this study, we will only consider $\lambda_{1}$, $\lambda_{2}$, and $t_{D}.$

We use the leakage factor relating the lower aquifer to the aquitard $\lambda_{2}=\sqrt{K_{2}\mathrm{Dc}}$ (L), ($\text{with}\;K_{2}$ [L/T] is the hydraulic conductivity, D [L] the thickness of the lower, pumped aquifer, and c [T] the resistance of aquitard) to define a dimensionless distance L:

(1) $$L=l_{A}/\lambda_{2}$$

where $l_{A}$ is the actual distance (L). Dimensionless time is defined as:

(2) $$t_{D}=\frac{K_{2}t}{S_{2}l^{2}}$$

with $K_{2}$ (L/T) being the hydraulic conductivity and $S_{2}$ (L^−1^) the specific storage of the pumped aquifer, *t* (T) is the actual time and l (L) is the distance from the pumping well.

Since the drawdown in the whole system is triggered by the drawdown in the pumped aquifer, we define the dimensionless drawdown as:

(3) $$s_{d}=\frac{K_{2}4\pi s}{Q}$$

where *s* (L) is the drawdown (L) and *Q* (L^3^/T) is the discharge of the pumping well (assumed to be constant in time).

Both the dimensionless time and drawdown are only based on properties of the pumped aquifer and the aquitard. We cover the properties of the other aquifer by testing the impact of hydraulic conductivity $K_{1}$ in different scenarios.

### Groundwater Flow Model

**Figure 2:**
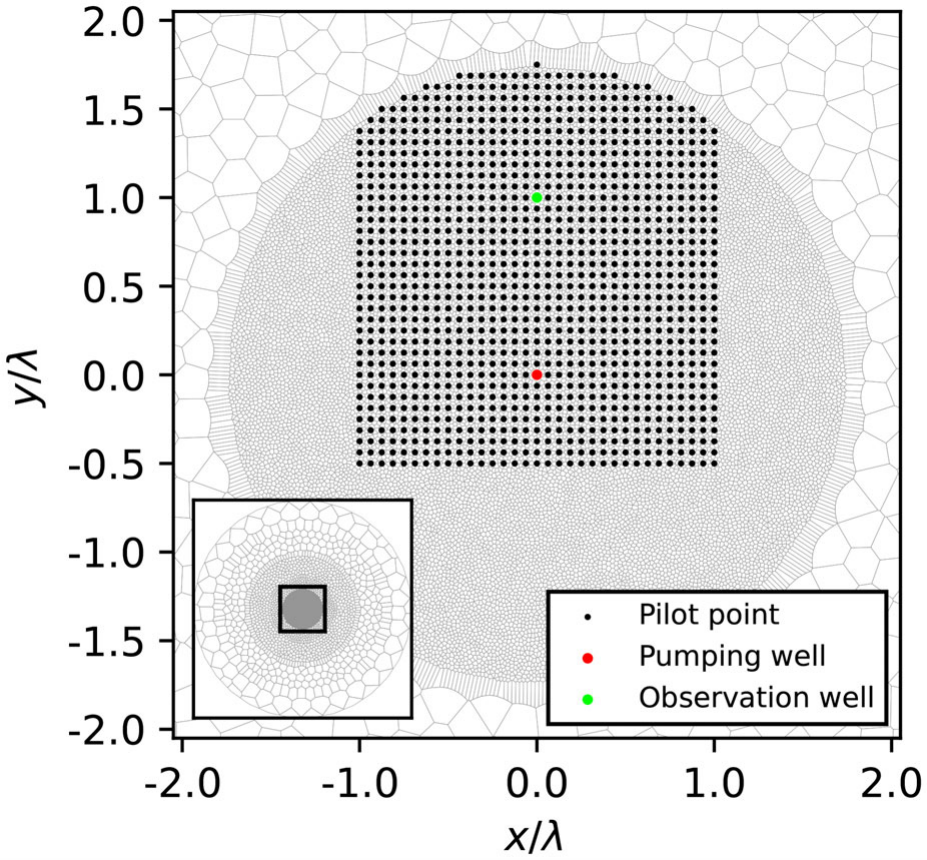
Model grid (top view) with pumping well, observation well, and pilot point locations. The inset shows the full grid.

The pumping test was simulated using the MODFLOW 6 model, created with FloPy (Bakker et al. 2016; Hughes et al. 2024). The 3D model contains three layers in the vertical direction and a circular Voronoi grid in the horizontal direction. This type of grid construction starts with cell centers, which are then transformed into grid cells using the Voronoi tessellation in FloPy, allowing for arbitrary cell shapes and sizes instead of creating the grid based on cell sizes beforehand, as is usual in structured grids. The grid is visualized in Figure 2 and is the same in all layers. There are approximately 22,000 grid cells in total. The smallest cell sizes are chosen so there are approximately 40 cells between the pumping and extraction well. The irregular grid allows for decreasing cell sizes toward the center. The circular shape of the grid is suitable for radial flow toward a well. Also, the central cell with the pumping well is a polygon that is approximately circular, which gives more accurate results locally than square grid cells.

The domain is large enough (L = 10, see Equation 1) to ensure boundary effects are minimized, similar to the infinite domain assumption from analytical solutions. The observation well is placed at L = 1 (one dimensionless distance) from the pumping well (Figure 2). It has screens in both aquifers (Figure 1). The MODFLOW model is validated with TTim (Bakker 2013) which provides analytical or semi‐analytical solutions for the same flow settings.

The initial head is set to 0 everywhere. The two top boundary settings are implemented separately. A no‐flow upper boundary is used for the confined top layer. The semiconfining top layer is simulated with a General Head Boundary with a head of 0 and a conductance that is equivalent to the hydraulic resistance of the aquitard.

Beside the two upper boundary settings, we test three scenarios for the relation of hydraulic conductivity/leakage factors in both aquifers: we test equal parameters $\lambda_{1}=\lambda_{2}$, a more conductive upper aquifer $\lambda_{1}=2\lambda_{2}$ and a more conductive lower (pumped) aquifer $\lambda_{1}=0.5\lambda_{2}$. Due to the square root relationship of leakage factor to hydraulic conductivity, *K*_1_ is varied by a factor 4. These values are selected as they are expected to change the flow pattern enough to investigate the effects of these parameters, while still remaining within realistic bounds. We only changed the transmissivity of the upper aquifer, as the solution will be independent on the transmissivity in the pumped aquifer, due to the conversion to dimensionless time and dimensionless drawdown (Equations 2 and 3). The influence of the specific storage has not been investigated and the value is kept constant in both aquifers. The three transmissivity scenarios (*K*_1_ > *K*_2_, *K*_1_ = *K*_2_, *K*_1_ < *K*_2_) in combination with the two boundary conditions (confined, semi‐confined) result in six scenarios in total.

### Sensitivity Analysis

We determine sensitivity maps of the aquitard for the head changes at the observation wells through perturbations at individual pilot points (Figure 2). The values of aquitard hydraulic conductivity *K* and specific storage S are systematically perturbed for each pilot point, and the changes in head are determined at the observation well. This way, the sensitivity is determined of the part of the aquitard influenced by each pilot point for the head in the observation well. The analysis is performed with PEST++ (White et al. 2020), specifically using the PESTPP‐GLM (Gauss–Levenberg–Marquardt) software and pyemu (version 1.3.8), the Python implementation of PEST++.

We chose to use pilot points instead of the grid cells for the perturbations because the fine grid resolution needed for accurate flow calculation is not necessary for the sensitivity maps. We placed the pilot points on a structured grid in the area surrounding the pumping and observation well (Figure 2). Points are L = 0.0625 apart, so there are 16 pilot points between the observation well and the pumping well.

We used default settings in PEST++/pyemu, meaning the perturbations of *K* and *S* were 1% of the log‐transformed initial values. We used a logarithmic transformation as *K* and *S* cannot be negative, but can cover multiple orders of magnitude. Sensitivity in this paper is defined as the absolute values of the Jacobian matrix, converted to dimensionless drawdown using Equation 3. This means it is a finite‐difference approximation of the derivative of the change in observation for hydraulic conductivity the sensitivity is defined by $\frac{∆s_{D}}{∆\mathrm{Log}\left(K\right)}$ (−), and for specific storage with and $\frac{∆s_{D}}{∆\mathrm{Log}\left(S\right)}$ (−). The sensitivities are converted to absolute values, meaning that high sensitivity zones represent areas that affect drawdown substantially, while low sensitivity zones represent areas that do not affect the drawdown. When the aquitard is considered heterogeneous, indirectly, any parameters that will be inversely computed from these drawdowns will also have this sensitivity pattern, and represent a spatial averaging of the layer parameters, weighted based on the sensitivity values.

## Results

### Model Validation

**Figure 3:**
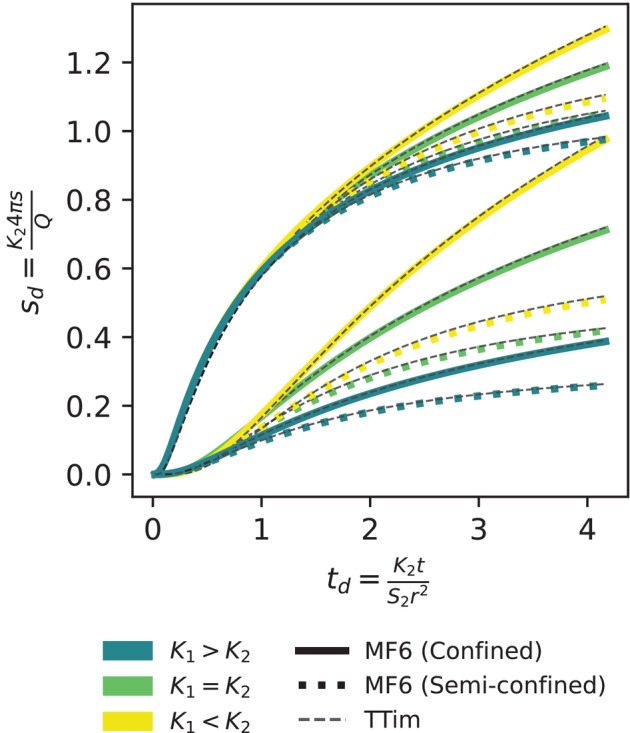
Pumping test drawdown at an observation well for three transmissivity scenarios (colors): Solid lines show the confined top boundary condition, while the dotted line represents the semi‐confined boundary condition. The dashed lines represent the TTim model results. The top curves represent the drawdown observed in the pumped aquifer; the bottom curves represent the drawdown observed in the overlying aquifer.

Figure 3 shows the drawdowns for all six scenarios for both the MODFLOW model and TTim simulations. The *R* ^2^ between the drawdowns at the observation wells of the two models is greater than 0.99 for each scenario. This confirms that the choices of grid and boundary conditions in the MODFLOW model are well suited for the analysis.

### Observation Well in Pumped Aquifer

**Figure 4:**
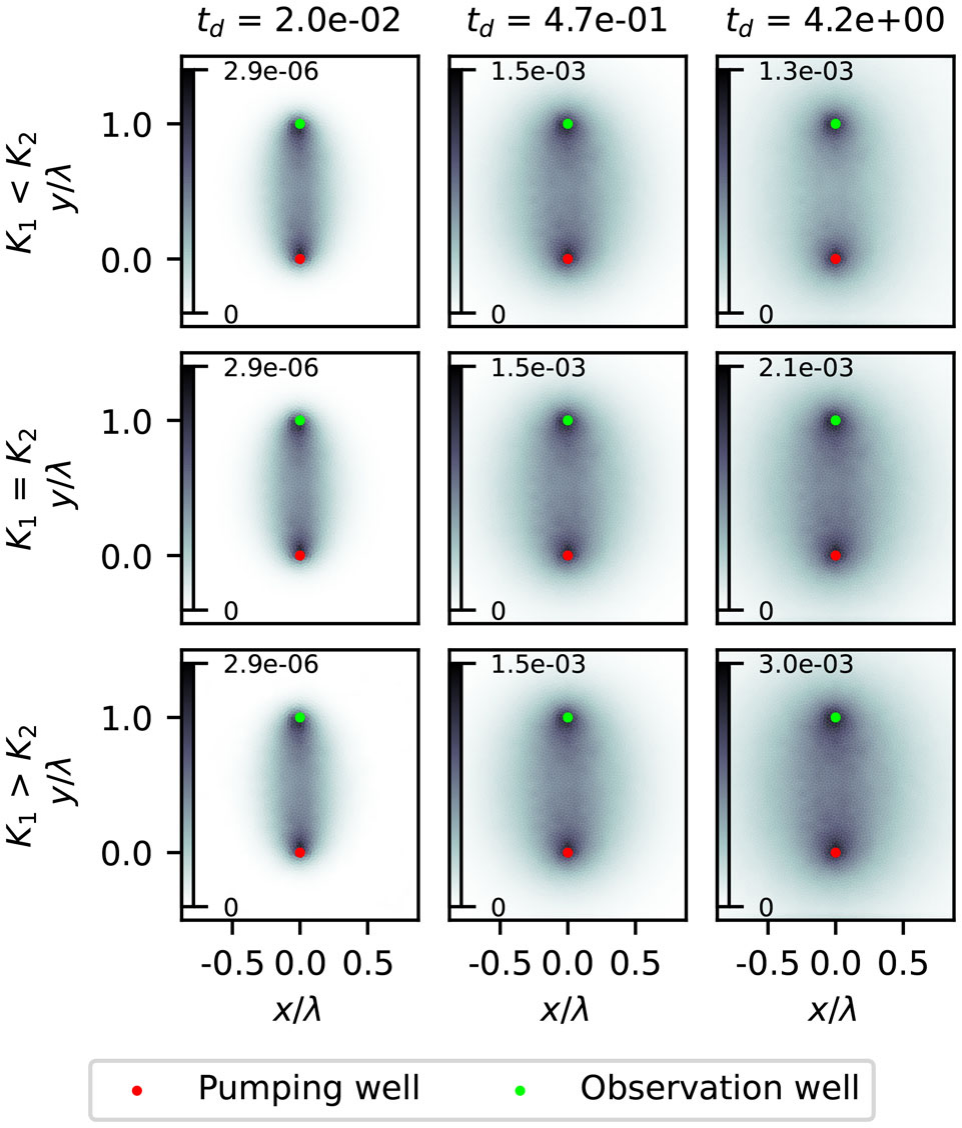
Sensitivity maps of aquitard hydraulic conductivity for drawdowns observed in the pumped aquifer with a confined top boundary. Sensitivity values are dimensionless. Dimensionless time increases from left to right. Top to bottom represents the three transmissivity scenarios.

Sensitivity maps of the aquitard resistance *c* for drawdown at the observation well in the pumped aquifer are shown in Figure 4 for the confined case. At early times, the sensitivity forms a narrow zone between both wells, with maxima at both wells and rapidly diminishing sensitivity away from the wells. Over time, the area of the sensitivity zone expands, while the maximum sensitivity values increases, except in the $K_{1}<K_{2}$ scenario, where the maximum value is slightly lower in the latest timestep than in the one before. In this scenario the signal propagates through the pumped aquifer first, meaning the drawdown here stabilizes quickly, reducing sensitivity. Generally, this affirms the notion that longer pumping provides more accurate results regarding aquitard hydraulic conductivity. While absolute sensitivity values are shown, the sign is the same throughout the whole domain before this conversion, which differs from the sensitivity pattern for hydraulic conductivity in aquifers, where the sign is different between the wells compared to outside of the wells (Leven and Dietrich 2006).

The spatial distribution is similar across all scenarios; only the exact values differ due to the difference in measured drawdown between the scenarios (Figure 3). Since the sensitivity pattern is the same, the top boundary condition does not affect the sensitivity of the observation well in the pumped aquifer. The sensitivity analysis for specific storage shows this pattern in all scenarios as well. The sensitivity maps of both the specific storage and hydraulic conductivity of the semiconfined case can be found in the Supporting Information S1–S3.

### Observation Well in Overlying Aquifer

**Figure 5:**
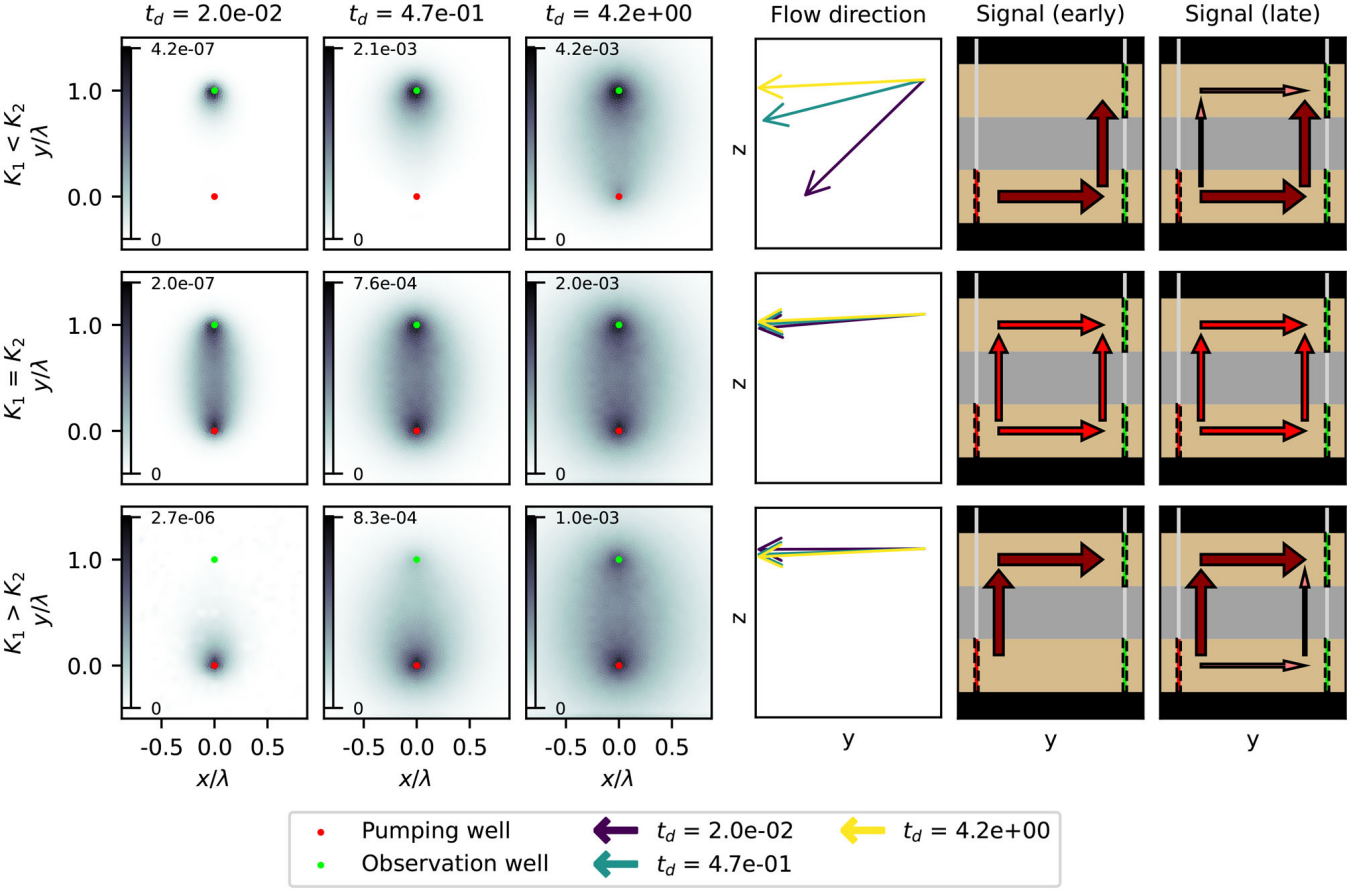
Left: Sensitivity maps of aquitard hydraulic conductivity for drawdowns observed in the overlying aquifer with a confined top boundary. Dimensionless time increases from left to right. Center: Flow direction in the observation well cell in the upper aquifer, showing the flow direction has a strong vertical component in the $K_{1}<K_{2}$ case for early time steps, shifting to horizontal toward late time steps. Right: Conceptual signal propagation for early and late times. Arrows represent the magnitude of the signal propagation from the pumping well to the observation well. Top to bottom are the three scenarios of hydraulic conductivity relation between the two aquifers.

The sensitivity maps of the aquitard hydraulic conductivity for the drawdown in the observation well in the overlying aquifer are shown in Figure 5 for the confined top layer scenarios. Moreover, Figure 5 shows flow directions over time and diagrams of signal propagation at early and late times. In contrast to the situation for the pumped aquifer, the sensitivity pattern of the observed drawdown in the overlying aquifer depends on the *K* values of both aquifers. When the *K* values are equal, the sensitivity pattern is like the one in Figure 4 for the observation in the pumped aquifer.

**Figure 6:**
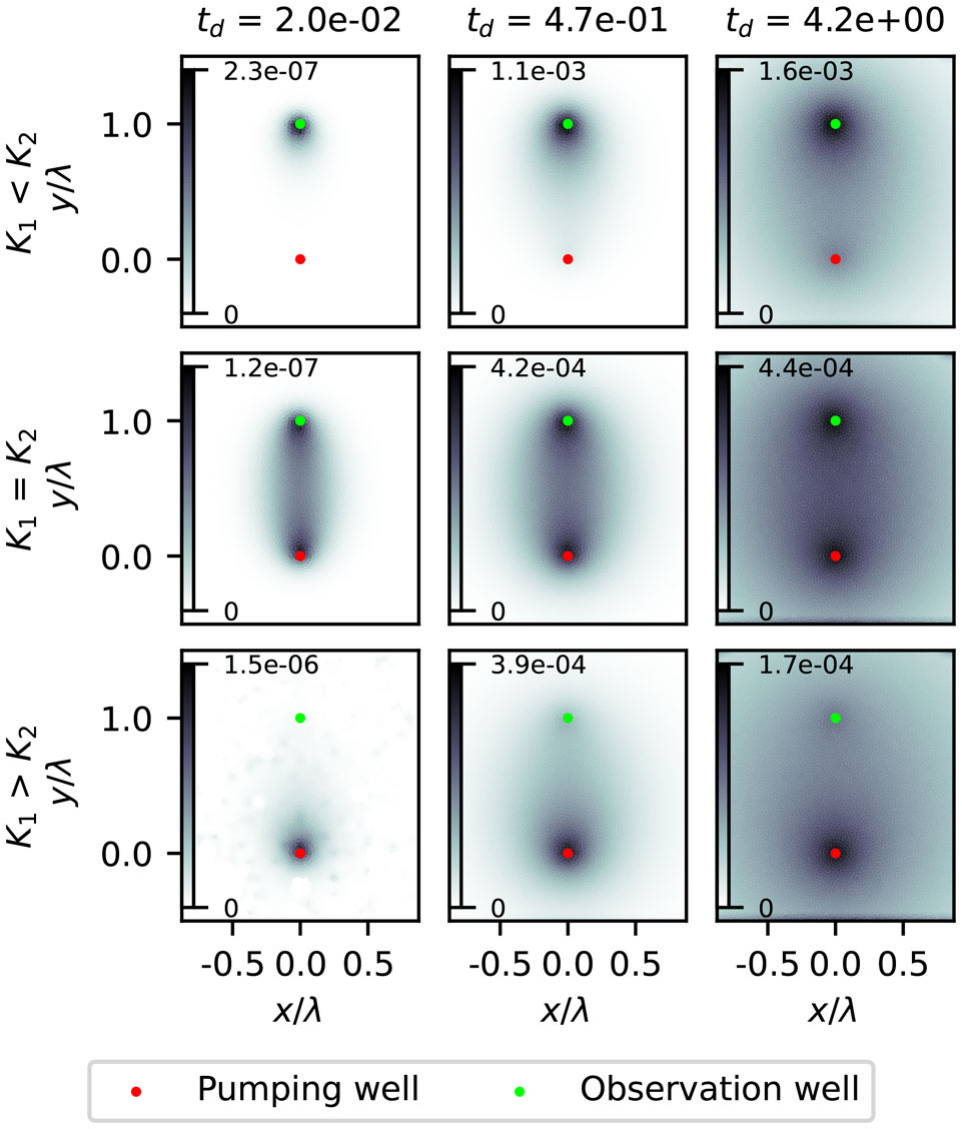
Sensitivity maps of aquitard specific storage for drawdowns observed in the pumped aquifer with a confined top boundary. Sensitivity values are dimensionless. Dimensionless time increases from left to right. Top to bottom represents the three transmissivity scenarios.

When *K* values differ between the aquifers, the sensitivity is concentrated at one of the two wells, especially at early times. The drawdown signal takes the route of least resistance, favoring the aquifer with the highest transmissivity (Figure 5). This pattern is confirmed by the change in time of the flow direction in the observation well; in the scenario where the hydraulic conductivity is higher in the pumped aquifer ($K_{1}<K_{2}$), the flow at the observation well initially has a large vertical component, shifting over time to a more horizontal direction. At later times, the sensitivity of the aquitard at the pumping well increases, indicating the signal eventually reaches the well through that route, but remains less strong. When $K_{1}>K_{2}$, the sensitivity starts at the pumping well and the flow direction is initially horizontal and shifts slightly downward later. Figure 6 shows the sensitivity maps for the specific storage for observation wells in the overlying aquifer. The main difference is that the magnitude of the sensitivity for specific storage is generally lower, meaning it affects drawdown less than hydraulic conductivity. Also, in the case of $K_{1}>K_{2}$ the maximum sensitivity is lowest in late timesteps, as opposed to the hydraulic conductivity pattern, were the maximum sensitivity is lowest in the $K_{1}<K_{2}$ scenario.

**Figure 7:**
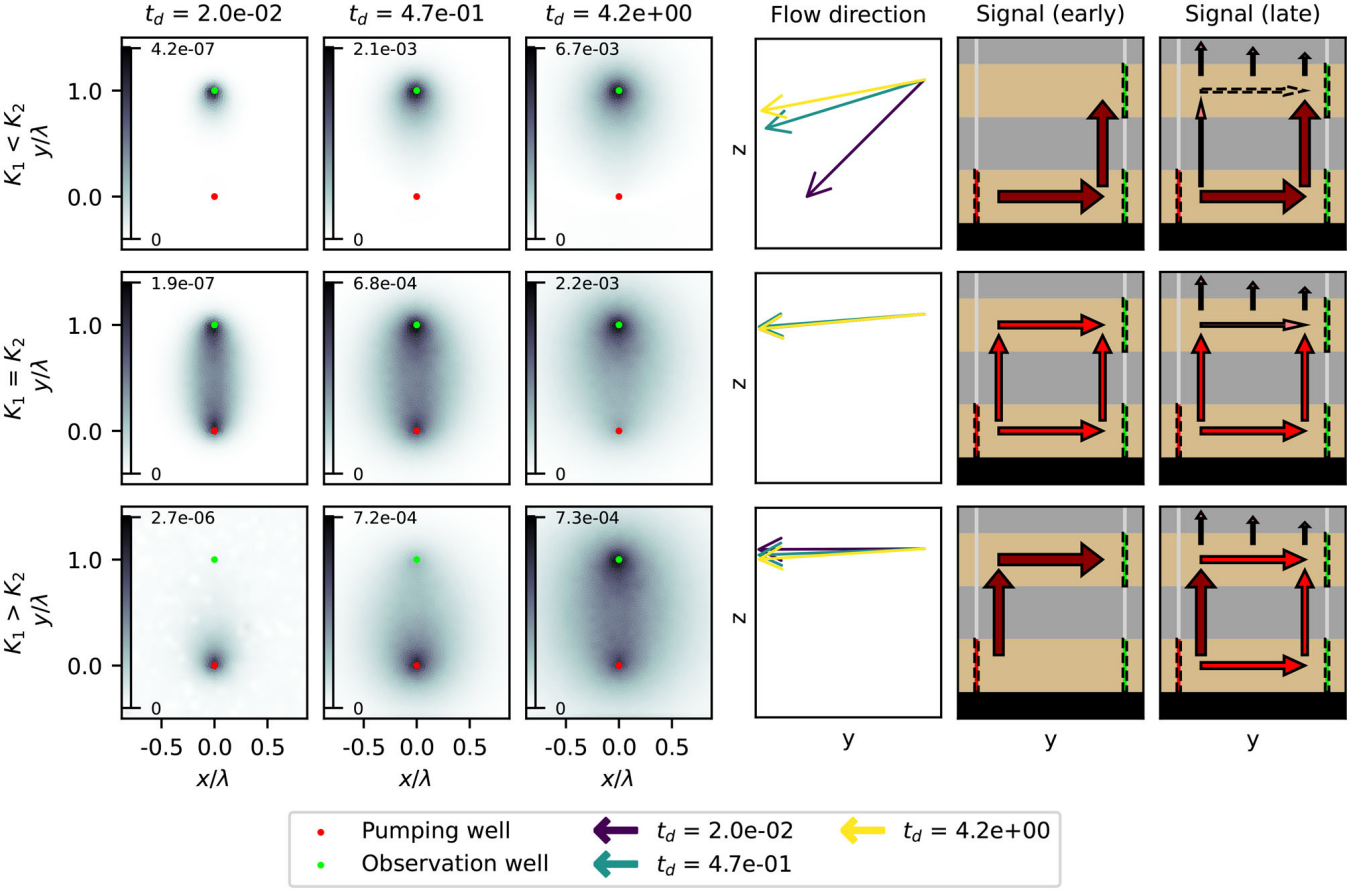
Left: Sensitivity maps of aquitard hydraulic conductivity for drawdowns observed in the overlying aquifer with a semiconfined top boundary. Dimensionless time increases from left to right. Center: Flow direction in the observation well cell in the upper aquifer, showing the flow direction has a strong vertical component in the $K_{1}<K_{2}$ case for early time steps, shifting to horizontal toward late time steps. Right: Conceptual signal propagation for early and late times. Arrows represent the magnitude of the signal propagation from the pumping well to the observation well. Top to bottom are the three scenarios of hydraulic conductivity relation between the two aquifers.

The hydraulic conductivity sensitivity maps for the semiconfined top boundary are shown in Figure 7. The general pattern is similar to the confined boundary case (Figure 5). With the semiconfined top boundary, the sensitivity shifts toward the observation well over time for all three scenarios. The flow vectors in the observation well have a larger vertical component compared to the confined case, especially at later times. This can be explained by the reduction of the drawdown signal through the overlying aquifer.

The sensitivity of specific storage shows the same pattern as the sensitivity of aquitard conductivity for both top boundary conditions. Sensitivity maps for the specific storage for the semi‐confined case are found in the Supporting Information S4.

## Discussion

### Limitations

This study shows the spatial sensitivity of drawdown to aquitard hydraulic conductivity and storage. This spatial sensitivity is only relevant when the aquitard cannot be assumed homogeneous, because otherwise it does not make a difference what part of the aquitard is represented in the drawdowns. The model layers are, however, homogeneous. The fact that the transmissivities of the aquifers affect the spatial sensitivity pattern by allowing the signal propagation to occur through the aquifer with the highest transmissivity suggests that within the aquitard the signal will also propagate through parts with the highest conductivity, at least in early timesteps. The magnitude of this effect, however, depends on how much these areas deviate from the mean hydraulic conductivity and whether these have the potential to affect the heads in both aquifers. That being said, it is likely that high conductivity regions in highly sensitive areas in this study will have a larger effect than in low sensitive areas. This makes the sensitivity maps still relevant for heterogeneous aquitards. There will probably be a level of variance in the aquitard where this is not the case anymore, when the effect of the heterogeneity will substantially affect the head distribution in all layers. In that case, the concept of aquifer and aquitard layers may not properly characterize the hydrogeological setting anymore.

Similarly, heterogeneous aquifers might also affect the sensitivity, as the sensitivity of the aquitard depends on the path with the lowest resistance. It can, however, be assumed that the relative transmissivities between aquifers remain a dominant factor as long as the aquifers have a hydraulic conductivity that is much larger than that of the aquitard.

In the scenarios, only *K* has been varied. It can be expected that the specific storage ratio between the aquifers also affects the drawdown propagation.

### Heterogeneity

Understanding the spatial sensitivity of aquitard properties for drawdowns in a pumping test is the first step to determine the heterogeneity of the aquitard, as the sensitivity pattern of the drawdowns also directly impacts the sensitive area of parameters inferred from these drawdowns. While identifying the structure of heterogeneity from observations at a single well is not straightforward based on the sensitivity patterns and their changes in time, it does provide an understanding of what potential information could be obtained from drawdowns. With multiple observation wells, interpreting each well separately at different time steps is an option to provide first insights into the heterogeneity in the aquitard. Differences between inferred parameters from various wells at different times provide insight into whether the aquitard needs to be considered heterogeneous at all. Model calibration is the next step to (re)construct a heterogeneous conductivity field or determine geostatistical parameters of heterogeneity, such as correlation length and variance. Designing model calibration to prevent non‐uniqueness is beyond the scope of this study, although placing pilot points in the aquitard at the locations of the wells is a good starting point, which is typically ill‐advised for aquifers (Doherty et al. 2011).

### Pumping Test Design

The results provide useful information for designing pumping tests focusing on aquitard characterization. Depending on the hydraulic conductivity of the aquifers, the spatial domain of the aquitard contributing to the drawdown varies. If pumping occurs in the more conductive aquifer, additional observation wells provide information about a unique part of the aquifer, especially with head observations in non‐pumped aquifers. In this case, there is value in installing a large number of observation wells. If the pumped aquifer is less conductive, the area around the pumping well is mainly characterized. Thus, observation wells in the overlying aquifer provide little additional information in this scenario, as all wells will be sensitive to the same region of the aquitard. In these cases, it might not be worth the expense of installing a large number of observation wells. While it can be challenging to estimate the conductivities of the aquifers before pumping, borehole descriptions might be sufficient to determine the approximate conductivity ratio between the aquifers.

Sensitivity patterns also evolve over time; in settings with a semiconfined upper boundary, the zone of peak sensitivity can shift from the pumping well toward observation wells. Extended pumping durations are therefore beneficial, not only to allow drawdown to propagate into non‐pumped aquifers, but also to capture the temporal shift in sensitivity. This means that a single observation well may inform resistance characteristics across multiple regions of the aquitard.

In practice, these temporal dynamics can take a significant amount of time. In this study, the shift occurred between dimensionless times of $t_{D}=0.47\;\text{and}\;t_{D}=4.2$ (Figure 7). Assuming the pumped aquifer has a hydraulic conductivity of 10 m/day, a specific storage of 0.0001 m^−1^ and the distance to the observation well is 1000 m, these dimensionless times correspond to 4.7 and 42 days (Equation 2).

## Conclusion

This study provides an analysis of the spatial sensitivity of aquitard hydraulic conductivity and specific storage observed in pumping tests as presented in sensitivity maps. By utilizing a three‐layered MODFLOW 6 model and performing a local sensitivity analysis with PEST++, we have demonstrated how spatial perturbations in aquitard hydraulic conductivity and specific storage affect head changes at observation wells in both pumped and overlying aquifers.

Our findings highlight the influence of aquifer conductivity on sensitivity patterns of aquitard parameters. For observation wells in the pumped aquifer, sensitivity is evenly divided between the observation well and the pumping well throughout time. In contrast, the sensitivity of parameters varies depending on the hydraulic conductivity contrast between the aquifers when observing in the overlying aquifer. The sensitivity is high in the area surrounding the pumping well when the pumped aquifer has lower conductivity than the overlying aquifer. When the pumped aquifer has a higher conductivity, the area surrounding the observation well has a large sensitivity.

The study also reveals how sensitivity patterns change over time, expanding the area of influence. In the case of a semiconfined top boundary, the sensitivity shifts toward the observation well. These results underscore the added value of running pumping tests for extended durations to capture this shift and obtain more information about the spatial heterogeneity of aquitard hydraulic parameters.

The sensitivity patterns obtained in this study can potentially be used to obtain heterogeneous conductivity fields and provide a basis for pumping test design with the objective of investigating aquitard heterogeneity.

## Authors' Note

The authors do not have any conflicts of interest or financial disclosures to report.

## Supporting information


**Data S1** Supporting Information.

## Data Availability

The code used in this study is available at https://github.com/MartijnVanLeer/PumpSens.
