# Supplementary figures and images for: Mapping the Spatial Sensitivity of Aquitard Hydraulic Parameters on Pumping Test Drawdowns
Source: Ground Water. 2025 Aug 25;64(1):41–8. doi: 10.1111/gwat.70014 (PMC12857531; doi:10.1111/gwat.70014)

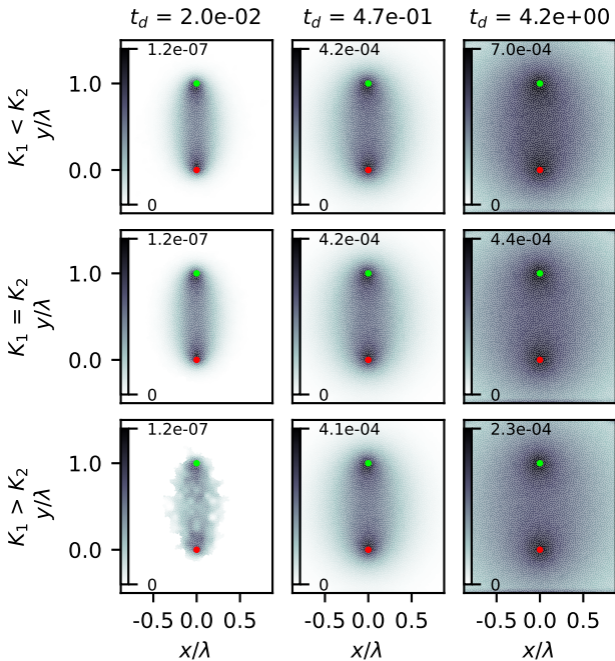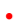

Pumping well

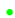

Observation well

Supplement: Supplementary file 1 — Data S1 Supporting Information. [file GWAT-64-41-s001.zip › gwat70014-sup-0002-Supinfo2.pdf]

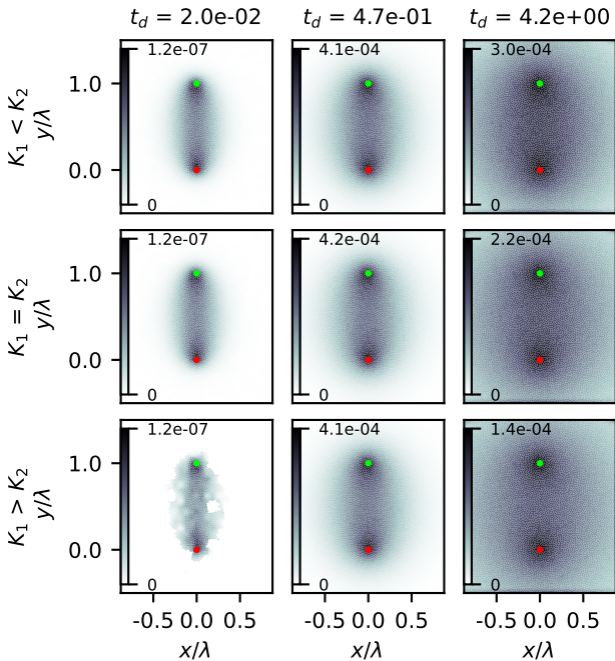

• Pumping well

• Observation well

Supplement: Supplementary file 1 — Data S1 Supporting Information. [file GWAT-64-41-s001.zip › gwat70014-sup-0003-Supinfo3.pdf]

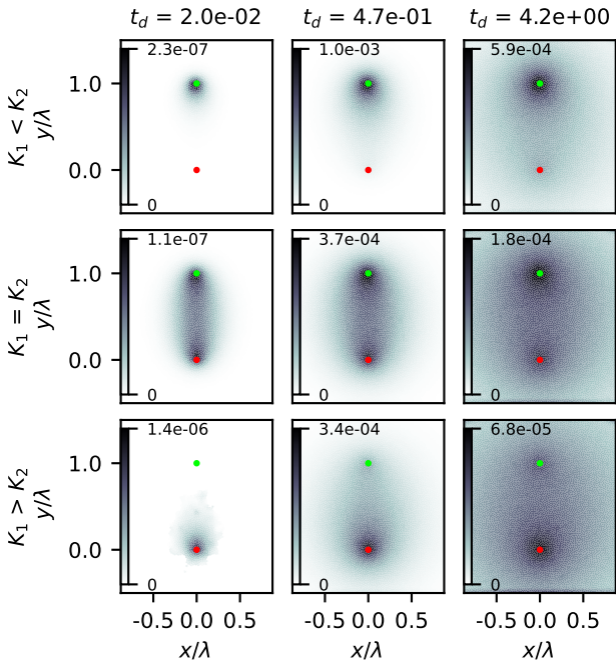

• Pumping well

• Observation well

Supplement: Supplementary file 1 — Data S1 Supporting Information. [file GWAT-64-41-s001.zip › gwat70014-sup-0004-Supinfo4.pdf]

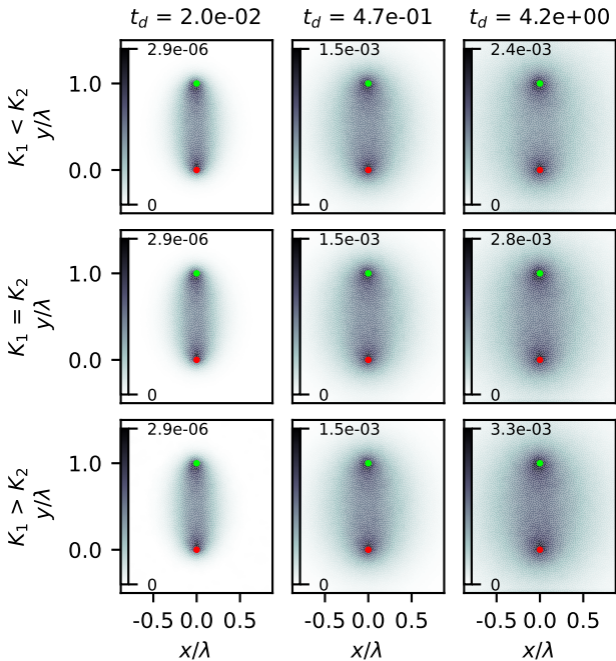

• Pumping well

• Observation well

Supplement: Supplementary file 1 — Data S1 Supporting Information. [file GWAT-64-41-s001.zip › gwat70014-sup-0001-Supinfo1.pdf]
